# Data Sharing During Pandemics: Reciprocity, Solidarity, and Limits to Obligations

**DOI:** 10.1007/s11673-023-10251-w

**Published:** 2023-07-13

**Authors:** Diego S. Silva, Maxwell J. Smith

**Affiliations:** 1 Sydney Health Ethics, School of Public Health, University of Sydney, Edward Ford Building, A27 Fisher Rd, Camperdown, New South Wales 2006 Australia; 2 School of Health Studies & Rotman Institute of Philosophy, Western University, 1151 Richmond Street, London, Ontario N6A 3K7 Canada

**Keywords:** Equity, Utility, COVID-19, Reciprocity, Solidarity

## Abstract

South Africa shared with the world the warning of a new strain of SARS-CoV2, Omicron, in November 2021. As a result, many high-income countries (HICs) instituted complete travel bans on persons leaving South Africa and other neighbouring countries. These bans were unnecessary from a scientific standpoint, and they ran counter to the International Health Regulations. In short, South Africa was penalized for sharing data. Data sharing during pandemics is commonly justified by appeals to solidarity. In this paper, we argue that solidarity is, at best, an aspirational ideal to work toward but that it cannot ground an obligation to share data. Instead, low-and-middle income countries (LIMCs) should be guided by the principle of reciprocity, which states that we ought to return good for good received. Reciprocity is necessarily a conditional principle. LMICs, we argue, should only share data during future pandemics on the condition that HICs provide enforceable assurances that the benefits of data sharing will be equitably distributed and that LMICs won’t be penalized for sharing information.

Of the ill-conceived policy decisions made during the COVID-19 pandemic, few were more ethically troubling than those made by many high-income countries (HICs) in response to Omicron in November 2021. When South Africa shared information about this novel SARS-CoV-2 variant with the World Health Organization (WHO), the country (and, inexplicably, many of its African neighbours) were met with travel bans. This response was ethically problematic in at least two ways: first, it was a policy decision based on racism and fear, not science, ethics, or law (Jackson, Habibi, Forman et al. 2022). These travel bans were enacted in contravention of the International Health Regulations (IHR) (World Health Organization 2016) and were resoundingly considered unnecessary by the scientific community (Mallapaty 2021; Mendelson et al. 2021; World Health Organization 2021). And second, it was grossly hypocritical of HICs to expect low- and middle-income countries (LMICs)^1^ to discharge their ethical and legal obligations (i.e., to promptly provide information about novel threats to the WHO), while ignoring their own obligations (e.g., to avoid unnecessary interference with international traffic and trade). As a result, not only was South Africa not made better off as a result of sharing this crucial data (i.e., fair benefit sharing), they were in fact made worse off.^2^

The treatment of South Africa and neighbouring countries in response to information shared about Omicron runs orthogonal to the declared commitments of politicians in HICs to solidarity. Part of this dissonance can be attributed to the fact that, more often than not, politicians used the concept of solidarity only rhetorically (Silva, Jackson, and Smith 2022). Yet, even substantive, normative arguments for data sharing^3^ grounded in solidarity seem to be divorced from the reality of global health. Cogent normative arguments for solidarity evidently hold little weight in guiding data sharing practices during an actual pandemic. Nevertheless, as of writing, the “conceptual zero draft” of the pandemic treaty currently being negotiated by the WHO and its member states centres the idea of solidarity and data sharing, arguing that we’ll need “…national, international, multilateral, bilateral, and multisectoral collaboration [including data sharing], coordination and cooperation in order to achieve a fairer, more equitable and better prepared world” (World Health Organization 2022, 12). Why should we think solidarity will serve us any better as a moral basis or guide for data sharing during future pandemics?

We argue that solidarity is likely to continue to function largely as a moral ideal, capable of generally motivating and inspiring the ways in which countries might decide to stand in relation to one another but incapable of guiding the concrete actions of countries as they relate to data sharing. We argue the principle of reciprocity is better suited to provide this sort of guidance. Articulating the moral demands and dimensions of data sharing in these terms is more likely to clarify what is morally required, permissible, and prohibited in this arena, and as a consequence, offers a guide for data sharing that is simultaneously more practical and more likely to address what we consider to be morally at stake when it comes to the sharing of data. As we shall argue, reciprocity emphasizes that insofar as LMICs have ethical obligations to share data with international authorities or HICs, these obligations are conditional upon receiving enforceable assurances that benefits will be shared fairly, and moreover, that there is no risk of being made worse off as a result of discharging these obligations.

## Solidarity and Reciprocity: Brief Descriptions

### Solidarity

Acknowledging that the concept of solidarity is contested and may admit of different interpretations in different areas of practice and scholarship, for the purposes of this paper we take solidarity to denote persons in positions of power centring the values and experiences of marginalized persons, while standing with them to promote just practices and policies. Solidarity can be understood relationally and “ … holds that individuals and communities are bound together, or mutually interdependent, and thus, that personal and collective well‐being are intimately linked” (Komparic, et al. 2019, 559). It can be understood as an intrinsic value that is foundationally good in-and-of-itself (Jennings and Dawson 2015) or as instrumentally valuable insofar as it promotes other values or norms (Prainsack and Buyx 2017), e.g., sharing vaccines means lowering everyone’s chances of catching nastier, mutated strains of a particular virus.

Generalist public health and global health literatures and policies commonly invoke solidarity as it relates to data sharing between HIC and LMICs. For instance, global health is replete with calls for solidarity as a moral basis for HICs to share various resources and technical expertise with LMICs.^4^ It is also commonly used, as we discuss in the next section, as a justification for data sharing for research and public health surveillance purposes, irrespective of the relative wealth or power of the states or regions in question.

### Reciprocity

In the public health ethics literature, reciprocity is most commonly invoked to justify compensation for someone who experiences losses in service of promoting public health. Two classic examples are owing compensation to those who undergo isolation because of an infectious disease (e.g., the provision of basic necessities for those isolating with pulmonary tuberculosis) or supporting healthcare workers who place themselves in danger for the common or public good (e.g., owing doctors and nurses personal protective equipment during outbreaks). As we outline below, it is sometimes—though less so—used as a means of justifying data sharing practices.

Whatever else is invoked or should be invoked by the concept of reciprocity, it means returning good for good received and compensating someone or some group for their losses in promoting someone else’s or some other group’s well-being (Upshur 2002). As Viens et al. note: “Reciprocity demands an appropriate balancing of the benefits and burdens of social cooperation necessary to obtain the good of public health” (Viens, Bensimon, and Upshur 2009, 211). And as a principle, reciprocity can be understood as

… a moral obligation involving an appropriate response by B commensurate with an action by A, where A’s action aims to contribute to, or bring about, public good, and where the action involves burdens, costs, or risks of harm to A. (Silva, Dawson, and Upshur 2016, 84)

Importantly, reciprocity does not entail, nor should it be used to justify, retribution or retaliation. As Becker notes, when discussing reciprocity in the context of virtue theory, retaliation is vicious precisely because it binds us to cycles of violence, broadly conceived (Becker 1990, 95). However, as Becker articulates and as the reader can deduce from the descriptions in the preceding paragraphs, reciprocity does not suggest or entail that *resistance* to another’s wrongdoing is morally wrong.

## Conditional Data Sharing: A Reciprocal Approach

As noted by Pratt and Bull in their recent literature review, maximizing utility is the primary driver behind calls for data sharing in health research and outbreak response (Pratt and Bull, 2021). In the context of an infectious disease outbreak or pandemic, one could argue that there’s an obligation to share data for surveillance and research purposes given (a) the severity of the situation, (b) the time constraints associated with responding to pandemics, and (c) because the knowledge gained usually cannot be ascertained at all or at a very high cost if data isn’t shared between jurisdictions and states. However, solely focusing on utility leaves us vulnerable to problems like the possibility of exploitation and the inequitable distribution of the benefits and burdens associated with data sharing (Pratt and Bull 2021).

Solidarity is another value commonly invoked to justify the obligations of states to share data during outbreaks and pandemics because it acknowledges utility while taking seriously questions of equity and fairness. In a paper we co-wrote with Langat et al. in 2011, we argued that “the health research community, as a community, is and ought to be regarded as united under a goal of combating disease” and that “embracing the value of solidarity highlights our common interests and shared vulnerabilities, providing additional impetus for collaboration in protecting human health and promoting human well-being” (Langat et al. 2011, 9). In short, we used solidarity as an ideal value to help justify an obligation to share data during pandemics. But note that we invoked shared humanity, common interests, and shared vulnerabilities; we wanted to promote data sharing for *everyone’s* benefit, thereby striving for a balance between utility (i.e., advancing the world’s capacity to understand and respond to infectious diseases) and equity (i.e., ensuring everyone benefits from the world’s capacity to understand and respond to infectious diseases). Solidarity is similarly deployed by the WHO in their ethics guidance documents in relation to both pandemic planning (World Health Organization 2007), as well as in response to COVID-19 (World Health Organization 2020). Meanwhile, McLennan and colleagues use the value of solidarity to argue that the European General Data Protection Regulation (GDPR) exemption should be invoked to facilitate data sharing during COVID-19 (McLennan, Anthony Celi, and Buyx 2020).

In turn, reciprocity is often used narrowly in the context of data sharing as a reason to support and protect individual or teams of researchers in sharing data, since they are in a position of risk when sharing. Research incentivizes secrecy—e.g., intellectual property to monetize products, the notion of “publish or perish,” etc.—so authors (again, including us) have previously deployed reciprocity as a means of articulating the need to think creatively about how to compensate and help those individuals who risk enjoying the full fruit of their research for the shared common interest of global health. However, writing in the context of the Ebola outbreak, the U.S. Presidential Commission for the Study of Bioethical Issues wrote that “reciprocity indicates that individuals and communities who have shared their biospecimens should have access to benefits that result from such research …. [especially] when dealing with samples collected from potentially vulnerable populations, including populations that are potentially vulnerable socioeconomically … ” (The Presidential Commission for the Study of Bioethical Issues, 2015, 47). Thus, the Presidential Commission correctly suggests that the risk of not benefiting from data sharing are not only borne by scientists but by their communities, too.

What public health ethicists (including us) have largely previously failed to imagine is not that those in positions of power would fail to share the benefits of data sharing with marginalized groups or states, i.e., being wronged via an omission, which was foreseen and is rather common. Instead, we failed to imagine the commissive prejudicial actions on the part of HICs and those in positions of power toward LMICs and marginalized communities as a *consequence* of data sharing with punitive results. It is particularly vile to take data shared by South Africa and use it to hinder it and other southern African countries. What makes the latter particularly morally problematic concerns the intention (or lack thereof) to abide by previously agreed to international rules, e.g., the International Health Regulations (World Health Organization 2016). HICs expect LMICs to abide by previously agreed to rules regarding pandemic response but repeatedly disregard whichever rules are inconvenient for them. Whatever other reasons exist to articulate why rules are morally important, it matters that following rules provides predictability and security that certain actions will follow in future instances should certain situations eventuate. Breaches of rules does away with predictability and the ability of agents to plan.

Given the realities of global health, namely, a backdrop of gross inequality and the fact that even during moments of extreme crises HICs appear incapable of acting fairly toward LMICs and global institutions, the use of solidarity increasingly rings hollow. The invocation of solidarity to morally justify data sharing assumes that all or most actors will acknowledge humanity’s shared vulnerabilities and common interests and then act on that basis. This assumption is false. In a non-ideal world that requires non-ideal norms and reasoning, solidarity is still valuable as lighthouse guiding ships in the fog. We should strive toward solidarity in global health, particularly as it relates to data sharing, but it largely fails to straightforwardly articulate the moral demands for data sharing, especially on the part of LMICs and others who are potentially marginalized.

Instead, data sharing should be guided by the principle of reciprocity. If reciprocity is about providing good for good received, then the onus is on HICs to demonstrate how (a) they will share the benefits of data sharing, and (b) will not take any actions that make LMICs worse off when they do share data. Data and samples are valuable; as such, it is morally permissible for LMICs to guard their dissemination and use unless and until politicians and governments in HICs can prove to them that they’re capable of playing nicely. No reasonable moral theory dictates that people must make themselves worse off when others have demonstrated repeatedly they are unwilling to abide by prearranged rules. Reciprocity has conditionality built into as a principle, whereas solidarity does not necessarily, which in the case of actors acting in bad faith, should be our moral starting point.

One could object that this will make addressing future pandemics more difficult with a *quid pro quo* attitude about data sharing, thereby reducing the utility associated with the practice. We would hope we could proceed without such arrangements, that solidarity would prevail and HICs would act accordingly. Our point is that there is little reason to believe this will be the case. Consequently, we may need to proceed on a *quid pro quo* basis.^5^ This is arguably going to make future pandemic responses more difficult for HICs. But it may make things more equitable. And efforts to advance equity may often require increased burdens on those in positions of power. On a simplistic understanding of utility and utilitarianism, we’ll bite the conceptual and normative bullet for the sake of equity. However, we’d argue that a more nuanced articulation of utilitarianism could still potentially make room for our conclusion, i.e., premising data sharing on the basis of reciprocity may have long-term overall better outcomes, while avoiding the potential deterrent effect of other countries hiding information given what happened to South Africa (Mallapaty 2021).

## Conclusion

There are at least two repercussions following from this argument: first, future iterations of the IHR and the WHO’s pandemic treaty need to reduce the place of prominence given to solidarity and instead introduce the principle of reciprocity as a moral guide for deliberations as it relates to data and information sharing. Second, although we focus our argument on the topic of data sharing, there’s a *prima facie* reason to believe it could also extend to other areas of collaboration during pandemics. Even during pandemics and other public health emergencies, LMICs who guard their assets and points of leverage closely and collaborate cautiously until they have seen tangible good-faith efforts on the part of HICs should be commended not derided, despite the clear importance of data sharing in such contexts.

## Footnotes

1 Recent global health literature (cf. Lencucha and Neupane 2022; Khan et al. 2022) has called into question the appropriateness of the use of “low-and-middle income country” as a term, whereby it reduces important differences to questions of economics and likely hides power imbalances. We use the term LMIC for lack of a better one, fully cognisant of its limitations.

2 Although perhaps most egregious, the Omicron incident is far from the only time that LMIC have been taken advantage of in the context of data sharing, where asymmetrical power relationships have meant pressures on LMIC to share samples despite no assurances of accessing the benefits of sharing (cf. Roos 2008; Freudenthal 2019).

3 For the sake of simplicity, by “data sharing” we also mean sharing of samples and results, though we acknowledge that different ethical issues may arise in sharing samples, data, and results. Regardless, solidarity is commonly invoked as justifying the necessity to share all three things.

4 In our observations, “technical expertise” is generally conceptualized as something that professionals from HIC give to those in LMIC, as if researchers and clinicians in LMIC don’t have technical expertise to share with HIC. This is slowly changing, we believe, but not quickly enough.

5 The *quid pro quo* here refers to the agent’s sharing of their best available data, not that the sharing of data must lead to the same amounts of benefits for all. We agree with Becker (1990) that “the purpose of reciprocity…. is to sustain mutually advantageous exchanges” (112). Ideally, there would be mutual and equal levels of benefit shared, but in reality this is often not the case; instead, what we can demand is equality of sacrifice (Becker 1990, 111–113). In the context of data sharing during pandemics, we can demand that countries share the best available data they have given their respective resources. Given the historical and present-day reality of colonialism in global health, *quid pro quo* can only mean equality of effort to share one’s best available data. A second reason we can only hold countries responsible for sharing best available data exists irrespective of economic resources: it seems likely that during a pandemic, some data shared will be more important at some given moments in time than others. For example, sharing data about a key viral mutation *x* that confers much greater morbidity than other mutations will mean that that the country (or countries) that found mutation *x* and shared that information will provide other countries with more benefit than they will receive by notifications of mutations that don’t result in an uptick of morbidity. As such, there are biological elements of luck regarding just how big or small the benefit of receiving data will be that are beyond the control of any one person or country.
