# Correction to: Data Sharing During Pandemics: Reciprocity, Solidarity, and Limits to Obligations

**DOI:** 10.1007/s11673-023-10307-x

**Published:** 2023-09-07

**Authors:** Diego S. Silva, Maxwell J. Smith

**Affiliations:** 1 Sydney Health Ethics, School of Public Health, University of Sydney, Edward Ford Building, A27 Fisher Rd, Camperdown, New South Wales 2006 Australia; 2 School of Health Studies & Rotman Institute of Philosophy, Western University, 1151 Richmond Street, London, Ontario N6A 3K7 Canada

**Correction to: Journal of Bioethical Inquiry**

**https://doi.org/10.1007/s11673-023-10251-w**

In this article the Conflict of Interests statement was missing and should have read as follows: 'The authors did not receive support from any organization for the submitted work.' The original article has been corrected.

